# Optimization of a mobile imaging system to aid in evaluating patients with oral lesions in a dental care setting

**DOI:** 10.1117/1.BIOS.2.4.042307

**Published:** 2025-10-06

**Authors:** Ruchika Mitbander, Jennifer Carns, Richard A. Schwarz, Sean Anderson, Geethanjali Vipulanandan, Miriam Hernandez, Amelia E. Hartzell, Jun Jiao, Ilona Rutkowska, Safia Durab, Sahar Sadeghi, Ashlee D. Clayton, Nancy Bass, Loganayaki Anandasivam, Rachel A. Giese, Ann M. Gillenwater, Nadarajah Vigneswaran, Rebecca Richards-Kortum

**Affiliations:** a Rice University, Department of Bioengineering, Houston, Texas, United States; b The University of Texas Health Science Center at Houston School of Dentistry, Department of Diagnostic and Biomedical Sciences, Houston, Texas, United States; c Harris Health Dental Center, Houston, Texas, United States; d The University of Texas Health Science Center at Houston School of Dentistry, UT Dentists, Houston, Texas, United States; e Brownsville Community Health Center, Brownsville, Texas, United States; f University of Texas Health Science Center San Antonio, Department of Otolaryngology-Head and Neck Surgery, San Antonio, Texas, United States; g The University of Texas M.D. Anderson Cancer Center, Department of Head and Neck Surgery, Houston, Texas, United States

**Keywords:** autofluorescence, oral cancer, early detection, imaging, machine learning, dental clinic

## Abstract

**Significance:**

Oral cancer is often diagnosed at an advanced stage even though the oral cavity is easily accessible for examination. There is a need for tools to support early detection and help non-expert clinicians make appropriate referral recommendations.

**Aim:**

We aim to evaluate a mobile detection of oral cancer (mDOC) smartphone-based autofluorescence imaging system by collecting data in a representative, low-prevalence population and optimizing a referral algorithm suitable for deployment in community dental settings.

**Approach:**

Data were collected from patients at a community dental clinic using the mDOC system, which captures white light and autofluorescence images. A multi-input deep learning algorithm was developed using both previously acquired and newly collected data in a rehearsal training method. Performance was evaluated on training, validation, and test sets using expert referral decision as the ground truth. Metrics included the Brier Skill Score, area under the ROC curve (AUC-ROC), sensitivity, and specificity.

**Results:**

Data from 252 anatomic sites across 50 patients were collected, with five sites in four patients referred to an oral cancer specialist based on expert review. The best-performing algorithm achieved a Brier Skill Score of 0.126. It reached an AUC-ROC of 0.986 on the validation set and an AUC-ROC of 0.778 on the test set. Sensitivity and specificity on the test set were 60.0% and 88.0%, respectively.

**Conclusions:**

These findings demonstrate the potential of the mDOC system and referral algorithm to assist in early detection and triage of oral mucosal lesions in low-prevalence, community dental clinic settings.

Statement of Discovery This work integrates white light and autofluorescence imaging with machine learning to develop a simple, low-cost smartphone imaging system for assessing oral lesions. Designed for use in community dental clinics, the system predicts whether a patient needs further evaluation by an oral cancer specialist. This technology has the potential to improve early diagnosis and enable timely, appropriate referrals to oral cancer specialists for improved patient outcomes.

## 1 Introduction

Early detection of oral cancer is critical for improving outcomes, when treatment is more effective.^1^^,^^2^ However, oral cancer is often detected at a late stage because early-stage lesions can appear benign and do not cause symptoms.^3^

The current standard of care for oral cancer detection is a conventional oral examination (COE), which involves visual examination accompanied by palpation under white light illumination. This method of screening is highly subjective, and accuracy suffers in part because the appearance of benign lesions can be similar to potentially malignant lesions and early cancer.^4^ There are conflicting recommendations for oral cancer screening. The American Dental Association recommends that a COE should be performed during routine dental visits.^5^ The U.S. Preventive Services Task Force does not recommend routine oral cancer screening by primary care physicians due to insufficient evidence for the benefits of oral cancer screening.^6^

Approximately 65% of adults in the United States visit a dentist annually, making it an ideal setting to conduct oral cancer screening regularly.^7^ Dentists and dental hygienists are often the first to encounter oral mucosal lesions and can play a vital role in detecting and managing benign oral conditions, oral precancers, and oral cancers. Yet, dental providers often lack the training and experience to recognize and distinguish oral precancers from benign conditions, which can lead to delayed referral to an oral cancer specialist and delays in diagnosis.^4^^,^^8^

Current oral cancer screening methods can be augmented with imaging tools to detect oral precancers and early cancers. Widefield autofluorescence imaging is one tool that has been extensively studied for oral cancer detection. Autofluorescence imaging relies on the use of blue light illumination and measures blue-green fluorescence. A loss in fluorescence is typically associated with neoplastic progression.^9^^,^^10^ Although this imaging method serves as a sensitive tool, it suffers in specificity, as many benign conditions, such as inflammation, are also associated with loss in fluorescence.^11^^–^^13^ In addition, many autofluorescence imaging tools rely on subjective interpretation of the results.

Deep learning techniques can offer a more objective method of interpreting results. Several studies have shown the promise of using deep learning combined with white light imaging^14^^–^^17^ and a few studies have also demonstrated the potential of deep learning to evaluate autofluorescence images acquired with mobile devices.^18^^,^^19^ Yet, few studies incorporate analyses based on expert referral recommendations for a range of oral conditions, including benign lesions. Thus, there remains a need for an objective and automated imaging tool to help triage patients in a dental care setting who need a referral for further evaluation by an oral cancer expert.

We developed a mobile detection of oral cancer (mDOC) device to aid clinicians in primary and dental care settings with the evaluation of patients with oral lesions.^20^ The mDOC device collects widefield white light and autofluorescence images of the oral cavity as well as oral cancer risk factors and uses machine learning methods to recommend whether patients should be referred for further evaluation by an oral cancer specialist. In a validation set with data acquired in two clinical settings consisting of a high-prevalence population, and data acquired in a healthy volunteer population, results showed that referral recommendations from mDOC had a sensitivity of 93.9% and a specificity of 79.3%, as compared with the expert referral decision.

The goal of this study was to optimize and assess the mDOC device and algorithm for referral management in a dental clinic setting, where the prevalence of oral lesions is low. mDOC was used to collect data from 50 subjects in dental clinic settings. Data were used to develop a multi-input mDOC referral algorithm that utilizes this dataset along with prior mDOC data to optimize algorithm performance for a more general dental clinic population. A rehearsal training method was used to retain learning from prior datasets, comprising a high-prevalence group and a healthy volunteer group, while evaluating the performance of the mDOC algorithm on a target deployment population, represented by the low-prevalence group in this study. The algorithm was used to classify oral sites for referral, using expert referral decisions as the ground truth.

## 2 Materials and Methods

### 2.1 Study Design

A cross-sectional pilot study of 50 patients presenting for routine periodic dental care or emergency dental treatment at UTHealth School of Dentistry at Houston and Harris Health Dental Clinic was conducted to evaluate the feasibility of oral lesion assessment using mDOC for referral management. The primary aim of this study was to develop the mDOC algorithm and evaluate its sensitivity and specificity for identifying patients requiring referral to an oral cancer specialist in a low-prevalence population. The mDOC referral recommendation was compared with that of a dental hygienist, dentist, and expert clinician.

Sample size calculations for the study were determined from power analyses with alpha=0.05, power = 0.80, and degrees of freedom =1. On this basis, a sample size of 32 was needed to detect large effect sizes, where a large effect size was defined as 0.5 for Cohen’s w in the power size calculation.^21^ A larger sample size of 50 was selected to allow for the possibility that images from some subjects may not be of sufficient quality for analysis.

### 2.2 Study Population

Patients who were 18 years of age or older and presenting for routine periodic dental care or emergency dental treatment at UTHealth School of Dentistry at Houston and Harris Health Dental Clinic were approached to participate in the study. Written informed consent was collected from all subjects. The study was conducted in accordance with recognized ethical guidelines and was reviewed and approved by the IRBs at Rice University (#2021-221) and UTHealth School of Dentistry at Houston (#HSC-DB-20-1318).

### 2.3 Imaging System

The mDOC device has been described previously.^20^ Briefly, it is a handheld smartphone-based imaging platform that captures white light and autofluorescence images of the oral cavity. At a working distance of 10 cm and its most commonly used zoom setting of 3× zoom, mDOC has a field of view of 2.5  cm×3.5  cm and 44  μm transverse resolution. A custom Android app guides the user to capture images of the oral cavity. The app can also record patient demographics (age, gender, ethnicity, and race) as well as patient smoking and drinking habits. The user records results of the COE, including a recommendation of whether to refer the patient for evaluation by an oral cancer specialist, before beginning the mDOC examination. mDOC is then used to capture one or more image(s) from sites with oral lesions identified on conventional examination and from up to five additional anatomic sites (including floor of mouth, right lateral tongue, left lateral tongue, right buccal mucosa, and left buccal mucosa). At each site, the capture button is pressed in sequence to initiate white light and autofluorescence imaging in under 3 s. The user is prompted to review image quality after each image pair is collected. Images can be retaken if needed. All images were uploaded to REDCap (RRID: SCR_003445).

### 2.4 Clinical Data Collection

#### 2.4.1 Clinical evaluation

The healthcare provider (dental hygienist or dentist) performed a COE. For each lesion, the clinician provided a recommendation of *Do Not Refer*, *Refer for Suspicion of Precancer/Cancer*, or *Refer for Reasons Other than Suspicion of Precancer/Cancer* (shortened to *Refer for Other*).

#### 2.4.2 mDOC imaging procedure

All clinical users were trained to use the mDOC device prior to imaging. Clinicians were trained to use the mDOC system on a custom training model consisting of an oral cavity with various oral mucosal lesions present. Each patient was evaluated and imaged with mDOC once by a dental hygienist and once by a dentist at their initial visit, and if referred, imaged again by an expert clinician at their referral visit. The duration of each imaging session was determined from the timestamps of the first and last image taken for a session.

#### 2.4.3 mDOC data review

mDOC images acquired by dental hygienists and dentists were reviewed by an expert clinician, including an oral pathologist and/or a head and neck surgeon (N.V. and/or A.G.) within two weeks of the patient’s initial visit. The review included patient demographics, patient-reported smoking and drinking habits, and image data, but expert clinicians were blinded to the initial clinical referral recommendations and the mDOC algorithm referral recommendation. Expert clinician(s) provided their referral recommendation based on this data review.

#### 2.4.4 Referral visit

Patients determined to need referral based on the initial provider or expert image review were scheduled for a referral visit to an oral cancer specialist within a month of their initial visit. At the referral visit, a conventional oral exam and mDOC exam were repeated by an expert clinician, and their referral recommendation for each anatomic site imaged was recorded. A biopsy was scheduled if clinically indicated.

### 2.5 Dataset Curation

A previous study^20^ developed an mDOC algorithm from data collected from 120 patients in a high-prevalence group who presented with a suspicious oral lesion and 29 healthy volunteers. The prior data set included images from 313 anatomic sites, with 143 sites in the *Do Not Refer* category and 170 sites in the *Refer* category. Here, we fine-tuned the referral algorithm by including new data from this study in a low-prevalence population to augment the prior training and validation sets and to compose a separate holdout test set. The final training and validation sets included data from this study and the prior study, whereas the final test set consisted of data only from this study (the target population).

**Fig. 1:**
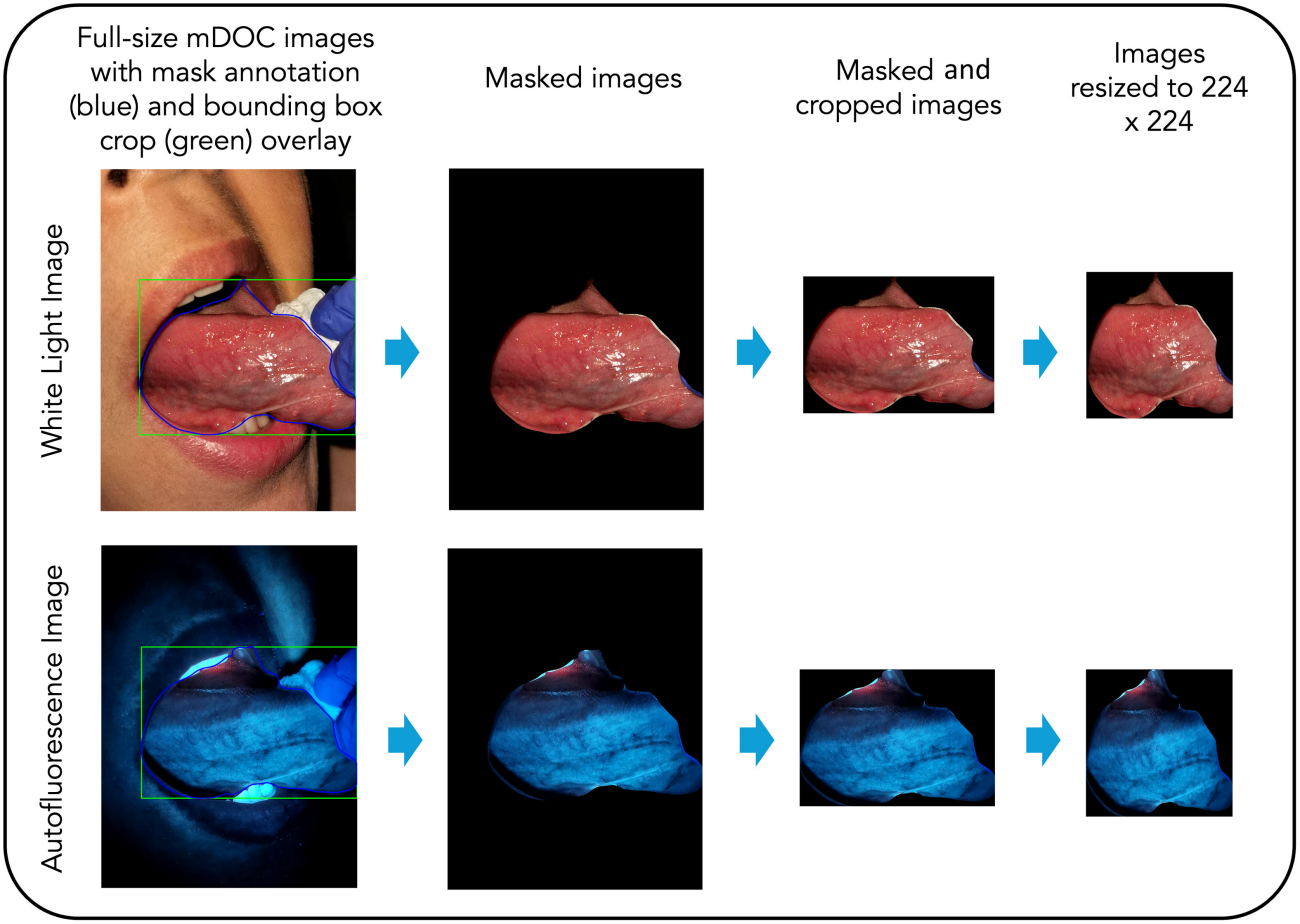
Data preprocessing workflow. Images are masked, cropped, and resized for image analysis.

A single reviewer manually assessed the quality of all white light and autofluorescence images collected. An image passed quality control (QC) if the reviewer judged that the anatomic site of interest was in focus and if at least 50% of the tissue of interest was illuminated. The reviewer was blinded to all clinical data associated with each image. Images that passed manual QC were annotated to outline the oral mucosa that included the primary anatomic site of interest and excluded teeth, gloves, dental tools, and background. Images were masked using the oral mucosa annotation and cropped to the bounding box of the oral mucosa annotation, then resized to 224×224 for subsequent image analysis (Fig. 1).

Oral cancer risk factors, including patient demographics, patient habits, and the anatomic site label, were also encoded for use as an additional input to the model. One-hot encoding, a strategy used to encode categorical data into a numerical format, was used for all categorical data, including race, gender, ethnicity, and for yes/no categories such as if the patient was a cigarette user, smoking tobacco user, smokeless tobacco user, or alcohol consumer. Patients with missing categorical data were assigned to an “unknown” category. Continuous data such as age were scaled using a minimum-maximum scaler. The average age was used to fill in missing age data to avoid introducing an additional feature for the missing value (n=1).

### 2.6 Multi-Input Classification Model

#### 2.6.1 Model inputs and outputs

A binary classification model was developed to identify patients with oral sites requiring further evaluation by an oral cancer specialist. The model inputs included mDOC white light and autofluorescence image pairs for the anatomic site and an oral cancer risk factor feature vector including patient risk factors such as demographics, smoking and drinking habits, and the anatomic site label associated with the image pair. The model output is a softmax probability that referral is recommended for an anatomic site. The ground truth label for each anatomic site was the expert referral decision based on expert review of mDOC data collected at the patient’s initial visit.

#### 2.6.2 Model architecture

**Fig. 2:**
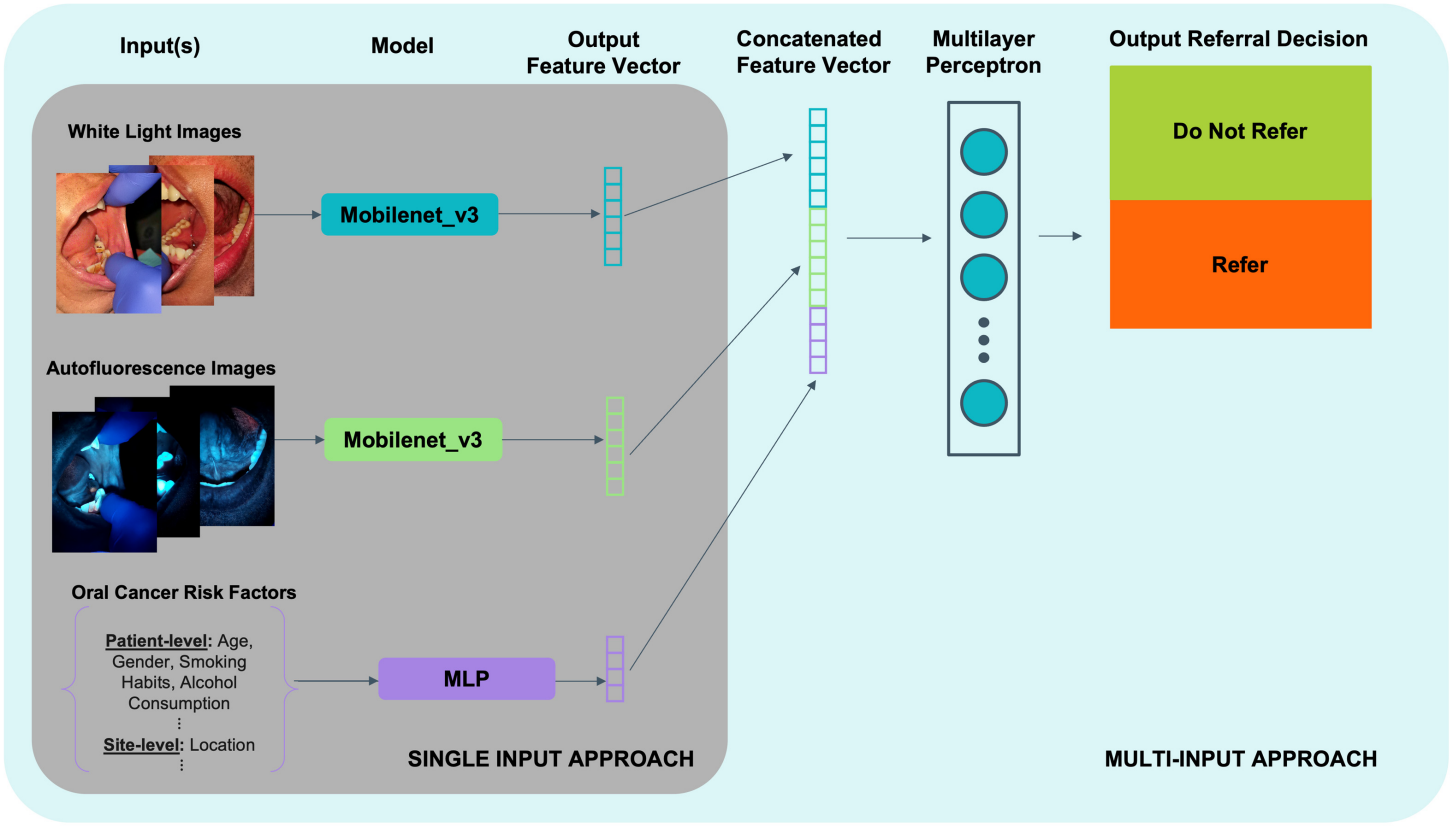
mDOC multi-input model architecture.

Figure 2 shows the model architecture developed to accommodate the use of image data and the oral cancer risk factor feature vector. The multi-input model was trained using both the image pair and the feature vector as inputs.

The multi-input model was composed of three individual models for each of the three inputs (WL image, AF image, and oral cancer risk factor feature vector). Each unimodal image model was built using a pretrained mobilenet_v3_small network as the backbone and was finetuned using a modified architecture. The classifier head of each individual unimodal model was modified to output 1024 features. A multilayer perceptron (MLP) with one input layer, one hidden layer, and one output layer was created for the oral cancer risk factor feature vector. The output feature vectors of each individual model were concatenated to create an intermediate input to the multi-input model classifier. A separate classifier head used the intermediate input that combined the outputs of the white light model, the autofluorescence model, and the feature vector MLP to generate the final referral decision output.

#### 2.6.3 Model development parameters

Training was set to a maximum of 120 epochs, with a 60-epoch minimum before stability was calculated. The training and validation batch sizes were set to five. Image standardization parameters, including the mean and standard deviation of image pixel intensity, were calculated using the white light and autofluorescence images in the rehearsal training dataset. These parameters were applied to the white light and autofluorescence images to standardize the images such that the mean was centered at zero with a standard deviation of one. The standardization was applied to the dataset with the other transforms during training, validation, and testing. Model training occurred on a computer with two GeForce RTX 2080 Ti graphics processing units, each with 11 GB SDRAM.

### 2.7 Rehearsal Method for Continual Learning

We leveraged learning on a dataset acquired from a group with a high prevalence of oral lesions and a healthy volunteer group and then updated the model with learning on the population of interest (a low-prevalence group). This method addresses a domain shift in the dataset based on the shift in prevalence of the training population and of the intended population for mDOC deployment. To prime the rehearsal model for training, a multi-input model was first developed on the prior dataset, described previously.^20^ A rehearsal model was initialized with these weights and then trained on the full dataset incorporating data from this study, with prior data shuffled in such that the model would experience replay of data it had previously trained on.

### 2.8 Classification Model Training, Validation, and Testing

#### 2.8.1 Priming model dataset partitioning

The data splits for the priming model used mDOC data collected in the high-prevalence and healthy volunteer dataset from the previously described study^20^ and were split into a training and validation set using an 80–20 split at a site level and stratified by referral decision.

#### 2.8.2 Rehearsal model dataset partitioning

The data splits for a rehearsal method used both prior mDOC data and data collected in this study. The prior data training and validation splits were maintained, and contralateral normal sites from patients who also had a lesion were dropped for the rehearsal stage. A separate holdout test set was composed of a subset of data collected only in this study, including data from all sites where the expert clinician recommended referral to an oral cancer specialist. The remaining subset of data collected in this study was split across the training and validation sets. This resulted in a final 52%-13%-35% training-validation-test split for the rehearsal dataset. Data splits were generated based on random grouped stratification, where all images from a single patient were grouped together and randomly assigned to either the training, validation, or test set, and the distribution within each dataset was additionally stratified by the ground truth expert referral recommendation.

#### 2.8.3 Metrics

The best-performing model was selected based on optimal loss curves and area under the receiver operating characteristic curve (AUC-ROC) performance for the validation set, and this model was applied to the test set. AUC-ROC was calculated using expert referral recommendation as the ground truth. The highest-scoring image was used for anatomic sites with multiple images for the AUC-ROC calculation. A Youden index metric was used to compute the best threshold, and sensitivity and specificity were calculated at this index value. In addition, a Brier score metric was calculated to assess the reliability of the model’s softmax probability output.

The GradCAM++ visualization method^22^ was modified for multi-input compatibility and used to generate model attention maps indicating regions of the image contributing to the final model output.

## 3 Results

### 3.1 Clinical Results

A total of 50 patients were enrolled in the study (Table 1). The study population was majority female (52%), white (32%), and not Hispanic or Latino (54%) with an average age of 49.2±15.1 years. In this study population, 14% of patients reported smoking cigarettes, 14% of patients reported using smoking tobacco, 4% of patients reported using smokeless tobacco, and 54% of patients reported drinking alcohol. All site-level referral decisions were *Do Not Refer* by the dentist or dental hygienist based on COE at the initial visit.

**Table 1:** Breakdown of study population characteristics.

		Patient study, N (%)
***N***		N=50
**Age**	Range (years)	20–87
	Mean (years)	49.2 ± 15.1
**Gender**	Female	26 (52.0%)
	Male	24 (48.0%)
**Ethnicity**	Hispanic	23 (46.0%)
	Not Hispanic	27 (54.0%)
**Race**	American Indian or Alaska Native	0 (0.0%)
	Asian	6 (12.0%)
	Black or African American	13 (26.0%)
	Native Hawaiian or other Pacific Islander	0 (0.0%)
	White	16 (32.0%)
	Not recorded	15 (30.0%)
**Cigarette smoking habits**	Smoker	7 (14.0%)
	Nonsmoker	43 (86.0%)
**Other smoking tobacco habits**	Smoker	7 (14.0%)
	Nonsmoker	43 (86.0%)
**Smokeless tobacco habits**	Smoker	2 (4.0%)
	Nonsmoker	48 (96.0%)
**Alcohol consumption habits**	Drinker	27 (54.0%)
	Nondrinker	23 (46.0%)

### 3.2 mDOC Imaging Results

Six clinicians were trained to use the mDOC device for imaging at the initial visit, including three dental hygienists, two dentists, and one expert clinician. A total of 252 anatomic sites were imaged with mDOC. All 50 patients completed imaging at their initial visit with the dental hygienist and dentist. The average time taken for each individual imaging session was 3.5±1.5  min for a typical imaging session consisting of imaging five standard anatomic sites with mDOC.

### 3.3 Referral Visit Results

Following expert review of mDOC data collected by dentists and dental hygienists, five anatomic sites, two of which came from a single patient, were recommended for referral to a specialist based on expert review. A total of four patients were referred to an oral cancer expert for further evaluation. The reasons cited for referral were *Refer for Other* for one patient and *Refer for Suspicion of Precancer/Cancer* for three patients. Three of the four patients attended the recommended visit for evaluation by an expert clinician and underwent COE and a repeat mDOC exam. One patient with a *Refer for Suspicion of Precancer/Cancer* decision did not return for a referral visit. Of the three patients seen at a referral visit, only one was recommended for biopsy based on COE. A visit was scheduled; however, the patient canceled their biopsy visit, and no biopsies were collected during the study period. The other two patients seen at a referral visit were determined not to require further evaluation based on COE because the sites of clinical concern based on expert image review had resolved by the time of the referral visit. No other patients had a clinically indicated biopsy taken as part of the study.

### 3.4 Dataset Curation for Rehearsal Training

Manual QC resulted in the removal of six imaged anatomic sites (24 images) from the dataset because they did not have a white light and autofluorescence image pair. The combined mDOC dataset included data from 516 anatomic sites, with 341 sites in the *Do Not Refer* category and 175 sites in the *Refer* category. The study-level breakdown of anatomic sites per referral decision for each of the training, validation, and holdout test datasets is shown in Table 2.

**Table 2:** Anatomic sites imaged with mDOC categorized by expert clinician referral decision for the training, validation, and holdout test datasets. *Refer for reasons other than suspicion of precancer/cancer.

Referral decision	Training population			Validation population			Test population	Dataset totals
	High-prevalence	Healthy volunteer	Low-prevalence	High-prevalence	Healthy volunteer	Low-prevalence	Low-prevalence	
**Do not refer**	1	79	51	0	20	15	175	341
Refer for other*	60	0	0	14	0	0	1	75
**Refer for suspicion of precancer/cancer**	77	0	0	19	0	0	4	100
***Study totals***	*138*	*79*	*51*	*33*	*20*	*15*	*180*	516
**Dataset totals**	268			68			180	

The training and validation datasets roughly had a 50:50 split of cases in which referral was recommended by expert review. The holdout test set was composed only of data from this study. There were 175 sites where referral was not indicated, and five sites where referral was recommended by expert review in the test set.

### 3.5 mDOC Priming Model Performance

The AUC-ROC of the multi-input model on the training set was 0.957, and the AUC-ROC for the validation set was 0.947. A Brier Skill Score for the model was 0.1145, a low score indicating that model prediction accuracy is good. This model was used to initialize the weights of the rehearsal model.

### 3.6 mDOC Rehearsal Model Performance

**Fig. 3:**
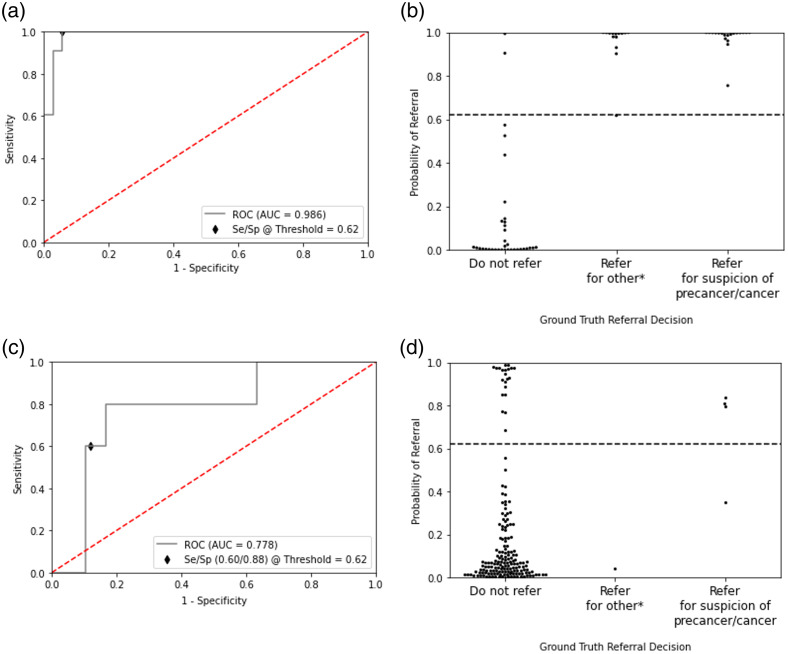
Rehearsal model performance. (a) ROC curve for model performance on validation set. (b) mDOC probability that referral is required for each site in the validation set, categorized by expert referral decision. (c) ROC curve for model performance on test set. (d) mDOC probability that referral is required for each site in the test set. *Refer for reasons other than suspicion of precancer/cancer.

The mDOC multi-input model trained using the rehearsal method was evaluated. The best performing model developed on image pairs in combination with the feature vector had an AUC-ROC of 0.998 on the training set, an AUC-ROC of 0.986 on the validation set, and achieved an AUC-ROC of 0.778 when evaluated on the holdout test set (Fig. 3). The Brier Skill Score was calculated to be 0.1260, a low score indicating more accurate model predictions. A threshold was calculated based on the validation dataset results using Youden’s index and applied to the test set. At this threshold, the sensitivity and specificity for data in the test set were 60.0% and 88.0%, respectively. Two sites recommended for referral by the expert clinician were incorrectly classified by the mDOC algorithm. In addition, 21 sites that did not require referral were misclassified as requiring referral [Fig. 3(d)]. Compared with recommendations made by the dental hygienist and dentist, which demonstrated 0.0% sensitivity and 100.0% specificity, the mDOC algorithm achieved higher sensitivity (60%) on the holdout test set, albeit with a modest decrease in specificity (88.0%).

**Fig. 4:**
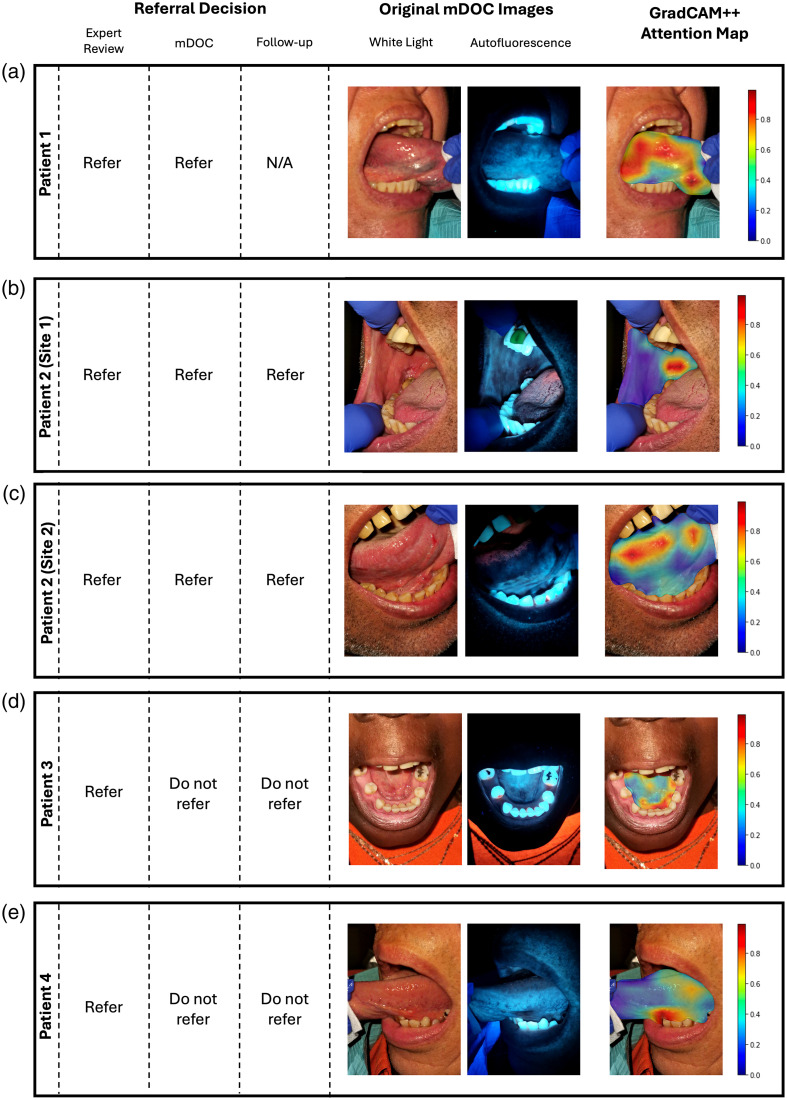
Sites recommended for referral by the expert clinician in the holdout test dataset. Left to right: referral decisions; mDOC white light (WL) and autofluorescence image pair acquired at the initial visit; GradCAM++ attention map overlaid on preprocessed WL image. (a) Right lateral tongue. Expert referral decision: *Refer for Suspicion of Precancer/Cancer*. mDOC referral decision: *Refer* (score = 0.81). (b) Right buccal mucosa. Expert referral decision: *Refer for Suspicion of Precancer/Cancer*. mDOC referral decision: *Refer* (score = 0.84). (c) Right lateral tongue. Expert referral decision: *Refer for Suspicion of Precancer/Cancer*. mDOC referral decision: *Refer* (score = 0.80). (d) Floor of the mouth. Expert referral decision: *Refer for Suspicion of Precancer/Cancer*. mDOC referral decision: *Do Not Refer* (score = 0.33). (e) Left lateral tongue. Expert referral decision: *Refer for Reasons Other than Suspicion of Precancer/Cancer*. mDOC referral decision: *Do Not Refer* (score = 0.04).

Five sites were recommended for referral by the expert clinician. Figure 4 shows results at each of these sites, along with the GradCAM++ attention map highlighting the regions contributing to the mDOC model prediction.

Figure 4(a) shows data from a suspicious lesion on the right lateral tongue. This site was deemed *Refer for Suspicion of Precancer/Cancer* by expert review. The mDOC referral decision was *Refer* with a score of 0.81, matching the expert referral decision. This patient did not return for a referral visit during the study period.

Figures 4(b) and 4(c) show data from two suspicious lesions in one patient, one on the right buccal mucosa [Fig. 4(b)] and one on the right lateral tongue [Fig. 4(c)]. Both sites were deemed *Refer for Suspicion of Precancer/Cancer* by expert review. The mDOC referral decision for both was also *Refer* with scores of 0.84 and 0.80, respectively. At the referral visit, a biopsy was clinically indicated for both sites; however, the patient did not schedule a biopsy visit during the study period.

Figure 4(d) shows data from a suspicious lesion on the floor of the mouth. This site was deemed *Refer for Suspicion of Precancer/Cancer* by expert review. The mDOC referral decision was *Do Not Refer* with a score of 0.33. At the referral visit, the lesion had resolved and did not require further evaluation. The mDOC referral decision based on images taken at the referral visit was *Do Not Refer* with a score of 0.18.

Figure 4(e) shows data from a suspicious lesion on the left lateral tongue. This site was deemed *Refer for Other* by expert review. The mDOC referral decision was *Do Not Refer* with a score of 0.04. At the referral visit, the lesion had resolved and did not require further evaluation. The mDOC referral decision based on images taken at the referral visit was *Do Not Refer* with a score of 0.32.

Overall, mDOC accurately classified three of the five sites recommended by experts for further evaluation. Although the two sites that were misclassified did not match the expert referral decision at the time of the initial visit, these lesions had resolved by the time of the referral visit.

## 4 Discussion and Conclusion

In this study, we collected data from a low-prevalence population of interest for mDOC deployment at UTHealth School of Dentistry at Houston and Harris Health Dental Clinic. We combined this data with a prior high-prevalence and healthy volunteer dataset and validated the performance of the mDOC referral algorithm for referral management in a representative low-prevalence test set.

We demonstrated that the updated mDOC model can generate referral predictions that generalize to a typical community dental clinic population. To support this, we utilized a rehearsal training method, which retains knowledge from previous datasets while allowing for incremental updates as new data are collected across diverse clinical sites. This approach may also accommodate changes in instrumentation over time. Model generalization remains a key challenge in medical applications due to variability in dataset collection procedures and differences in disease prevalence between the training datasets and the intended deployment population. It is common to observe a decline in model performance when transitioning from training and validation phases to field implementation. Therefore, it is critical to construct a training dataset with appropriate diversity and to evaluate it on a representative test set that mirrors the target use population. A strength of our study is the use of a representative holdout test set for model evaluation, composed of 97% non-referral (*Do Not Refer*) sites and 3% referral sites. This distribution aligns with the estimated 1% to 5% prevalence of oral potentially malignant disorders in the general population.^23^ On this test set, the mDOC algorithm achieved an AUC-ROC of 77.8%. At an operating threshold of 0.62, the sensitivity and specificity of the mDOC algorithm on the test set were 60.0% and 88.0%, respectively. A limitation of this analysis is the small number of referral sites (5 out of 180), reflecting the low-prevalence nature of the study population. Another limitation is the absence of histologically confirmed endpoints, which would provide a more definitive ground truth for model evaluation.

Despite the limited number of referral cases, the mDOC algorithm correctly classified three out of the five *Refer* sites based on expert review of the initial imaging session. As shown in Figs. 4(a)–4(c), the mDOC referral decisions matched those of the expert reviewer, demonstrating the system’s potential to support referral management in a general population. By contrast, Figs. 4(d)–4(e) illustrate two cases where the mDOC algorithm classified the sites as *Do Not Refer*, whereas the expert initially recommended referral. Notably, by the time of the specialist visit, the suspicious areas had resolved, and the final expert decision was changed to *Do Not Refer*. In these cases, mDOC may have correctly predicted that a referral was unnecessary, highlighting its potential to reduce avoidable referrals. However, of the 175 *Do Not Refer* cases, 21 were incorrectly classified by mDOC as *Refer*, resulting in 14 false positives across patients. These findings underscore the need for further refinement of the algorithm to improve specificity in low-prevalence populations. Additional methods such as data augmentation can be explored to build a more robust model. Collecting additional oral cancer risk factors such as a history of cancer and a history of oral cancer and including specific habit details such as smoking/drinking type and frequency could be incorporated into a future model.

In our current study, mDOC was used by six different clinicians. The average time to conduct the mDOC examination was 3.5 min across all providers. This is a reasonable amount of time to integrate within existing workflows, and with the addition of a near-real-time implementation of the model on the mDOC device, mDOC has the potential to aid in the detection of oral conditions that require further evaluation by an oral specialist in a timely manner.

## Data Availability

The data that support the major findings of this article are publicly available at https://gitlab.com/rrk-lab/public/mdoc-dental-clinic.
